# Anthropometric and metabolic indices in assessment of type and severity of dyslipidemia

**DOI:** 10.1186/s40101-017-0134-x

**Published:** 2017-02-28

**Authors:** Muhammad Zaid, Fatima Ameer, Rimsha Munir, Rida Rashid, Nimrah Farooq, Shahida Hasnain, Nousheen Zaidi

**Affiliations:** Microbiology and Molecular Genetics, University of the Punjab, Lahore, 54590 Pakistan

**Keywords:** Dyslipidemia, Plasma lipids, Body indices

## Abstract

**Background:**

It has been shown that obesity is associated with increased rates of dyslipidemia. The present work revisits the association between plasma lipid levels and classical indicators of obesity including body mass index (BMI). The significance of various anthropometric/metabolic variables in clinical assessment of type and severity of dyslipidemia was also determined. Recently described body indices, a body shape index (ABSI) and body roundness index (BRI), were also assessed in this context.

**Methods:**

For the present cross-sectional analytical study, the participants (*n* = 275) were recruited from the patients visiting different health camps. Participants were anthropometrically measured and interviewed, and their fasting intravenous blood was collected. Plasma lipid levels were accordingly determined.

**Results:**

The values for different anthropometric parameters are significantly different between dyslipidemic and non-dyslipidemic participants. Receiver operating characteristics curve analyses revealed that all the tested variables gave the highest area under the curve (AUC) values for predicting hypertriglyceridemia in comparison to other plasma lipid abnormalities. BRI gave slightly higher AUC values in predicting different forms of dyslipidemia in comparison to BMI, whereas ABSI gave very low values.

**Conclusions:**

Several anthropometric/metabolic indices display increased predictive capabilities for detecting hypertriglyceridemia in comparison to any other form of plasma lipid disorders. The capacity of BRI to predict dyslipidemia was comparable but not superior to the classical indicators of obesity, whereas ABSI could not detect dyslipidemia.

**Electronic supplementary material:**

The online version of this article (doi:10.1186/s40101-017-0134-x) contains supplementary material, which is available to authorized users.

## Introduction

Obesity is previously shown to be associated with increased rates of dyslipidemia and other cardiovascular risk factors [1]. Most of the previous works studied the association of dyslipidemia with body mass index (BMI) and waist circumference (WC) [2–6], which are commonly considered as the valid measures of obesity [7]. Nevertheless, several previous works have doubted the reliability of BMI as an indicator of obesity [8, 9]. It was reported that BMI may provide a false diagnosis of body fatness [8, 9], and it cannot distinguish between adipose tissue and lean body mass [10–14]. Moreover, it is observed that in young healthy adults, BMI and other surrogate indices of fatness (e.g., waist-to-height ratio, body adiposity index) provide poor prognosis of fat mass since they reflect mostly skeletal muscle mass [15, 16]. WC was shown to be a good predictor for abdominal adipose tissue [17, 18]. Nevertheless, it is unclear that to what extent the range of WC depends on body size [19]. Therefore, it was suggested that more appropriate body indices should be designed that will also take body shape into account and may serve as improved indicators of obesity [20–23]. These indices were suggested to be designed by combining traditional anthropometric measures, e.g., height, weight, BMI, or WC. In accordance with that, at least two new body indices have been developed—a body shape index (ABSI) [21] and body roundness index (BRI) [22].

ABSI is an index of body composition based on waist circumference, BMI, and height [21]. It has been suggested that high ABSI relates to a greater fraction of abdominal adipose tissue and appears to be a significant risk factor for premature death [21]. It has also been reported that ABSI could be used to evaluate physical health status of adolescents [24]. Previous studies indicated that ABSI is able to predict the new onset or presence of diabetes mellitus [25, 26]. However, the predictive capability of ABSI for diabetes mellitus was not better than that of WC and BMI. Moreover, ABSI is reported to be a weaker predictor of cardiovascular diseases (CVD) when compared to BMI [27].

Body roundness index (BRI), developed by Thomas et al. in 2013 [22], combines height and waist circumference to predict the percentage of body fat. This approach allows estimation of the shape of the human body figure as an ellipse or oval. The values for BRI range from 1 to 16, and rounder individuals tend to have larger values. Only few studies have determined the significance of BRI as an indicator of obesity. It has been reported that BRI could predict the presence of cardiovascular diseases (CVD) [27]. However, the predictive capability of BRI for CVD was not better than the established anthropometric indices like BMI and WC. In addition, the predictive capability of BRI for diabetes mellitus was comparable to that of BMI [25].

Several studies provide inconclusive data on association of plasma lipid levels with classical indicators of obesity including BMI. Few studies have shown that overweight and obese adolescents display higher levels of serum cholesterol, low-density lipoprotein cholesterol (LDL), and triglycerides (TGs) in comparison to the subjects with normal weight [28]. It has been previously reported that BMI and WC are positively correlated with serum cholesterol, LDL, and TG levels [2–6], whereas negatively correlated with serum high-density lipoprotein cholesterol (HDL) levels [2–6]. The present work aims to revisit the association between plasma lipid levels and various anthropometric/metabolic variables in a group of participants from urban and rural population of Punjab, Pakistan. This study examines in detail the significance of various anthropometric/metabolic variables in assessment of dyslipidemia. We have also compared the significance of the recently described body indices, ABSI and BRI, with the classical indicators of obesity. In addition to that, the effects of patterns and severity of dyslipidemia on various anthropometric and metabolic variables were determined. The predictive capabilities of the anthropometric/metabolic parameters for identification of various forms of dyslipidemias were also assessed.

## Materials and methods

### Participants, study protocols, and ethics

For the present cross-sectional analytical study, the participants (*n* = 275) were recruited from the individuals that visited 1-day free health camps organized by the Metabolism Group, Department of Microbiology and Molecular Genetics, University of the Punjab, Lahore. These free health camps were organized at the University of the Punjab and Nishat Colony Lahore; Jhokan village, Jhang; and Chack 306 Botala, Gojra during a period of 6 months from March 2015 to September 2015. The residents of the respective localities were invited for a free health examination. Informed consent was obtained from each participant before the sample collection. In order to obtain the basic personal information and medical history, each participant was interviewed and completed a structured questionnaire. The study was performed according to the Helsinki Declaration. The study protocol was approved by the Ethics Committee of School of Biological Sciences, University of the Punjab. The subjects with the following conditions were excluded: severe viral/bacterial infection, on anticoagulation therapy, suffering from bleeding disorder (e.g., hemophilia, low platelets), and aplastic anemia. In addition to that, participants that had history of diabetes mellitus, cardiovascular disease, or any cancer were also not included in the study population because these conditions could also interfere with plasma lipid levels [29]. The statin-users were also excluded from the study on the same grounds. For the final analysis, we included 149 dyslipidemic and 89 non-dyslipidemic participants (Additional file 1: Figure S1).

### Assessment of different anthropometric and metabolic parameters

Body weight was measured using standard analog weighing scale to the nearest kilogram. The measurements for height, waist circumference, hip girth, and wrist circumference were taken to the nearest 0.5 cm using a non-stretchable measuring tape. All of these measurements were taken without shoes, sweater, and jackets. The waist circumference (WC) was measured in the midway section between the iliac crests and costal margins at minimal respiration. The hip girth measurements were taken at the level of the greatest protrusion of the buttock muscles while the wrist circumference was measured around the widest point. The height, waist circumference, and hip girth measurements were used to calculate waist-to-hip and waist-to-height ratios. Systolic and diastolic blood pressure was measured according to the recommended techniques using digital sphygmomanometer. Fasting plasma glucose levels were spectrophotometrically determined using commercially available kit (Glucose-Liquizyme GOD-PAP Spectrum). Resting metabolic rate (RMR) was calculated using Mifflin-St. Jeor equations. Body fat percentage (BF %), total body fat mass, BMI, ABSI, and BRI were calculated according to standardized formulas as:

$$ \mathrm{Body}\ \mathrm{Mass}\ \mathrm{Index} = \raisebox{1ex}{$\mathrm{Weight}$}\!\left/ \!\raisebox{-1ex}{${\mathrm{Height}}^2$}\right. $$ [30]

body fat percentage (BF %) = (1.2 × BMI) + (0.23 × age) ‐ 5.4 [31]

total body fat mass = BF %/100 × body weight (Kg) [31]

$$ \mathrm{ABSI} = \frac{\mathrm{Waist}\ \mathrm{Circumference}\ \left(\mathrm{WC}\right)}{{\mathrm{BMI}}^{\frac{2}{3}} \times {\mathrm{Height}}^{\frac{1}{2}}} $$ [21]

$$ \mathrm{B}\mathrm{R}\mathrm{I}=364.2-365.5 \times \sqrt{1-\left(\frac{\left(\mathrm{WC}/{\left(2\uppi \right)}^2\right)}{{\left(0.5\times \mathrm{Height}\right)}^2}\right)} $$ [22]

Clinical and metabolic characteristics of the study population are depicted in Additional file 2: Table S1. Significant differences were observed between dyslipidemic and non-dyslipidemic participants for multiple anthropometric/metabolic indices.

### Blood sample collection

Intravenous blood was collected from all the subjects after 10 ± 2 h of fasting according to the guidelines of the National Committee for Clinical Laboratory Standards (H18-A4) [32] in vials containing EDTA. All samples were processed within 30 min of collection. Plasma was promptly separated.

### Determination of plasma lipid levels

Plasma total cholesterol (TC) and triglyceride levels were spectrophotometrically determined using commercially available kits (Analyticon Biotechnologies AG, 4046 and 5052, respectively). For the estimation of high-density lipoprotein cholesterol, other lipoprotein fractions were precipitated using HDL precipitation reagent (Analyticon Biotechnologies AG, 410). HDL was then estimated using the aforementioned Analyticon kit for the quantitative determination of cholesterol. For the estimation of low-density lipoprotein cholesterol, we used the recently described method by Martin et al. [33]. Lipoprotein abnormality status was determined by NCEP, Adult Treatment Panel III (ATP III) guidelines (Additional file 2: Table S2). Dyslipidemia was defined as irregularity in the plasma levels of at least one of the major lipoprotein fractions. Additional file 1: Figure S2 shows a Venn-Diagram that displays the overlaps between prevalence of high-LDL, TG, and low-HDL levels in our dyslipidemic population.

### Statistical analysis

The results were analyzed by Student’s *t* test using Graph-Pad Prism VI Software. *P* values <0.05 were considered statistically significant. Correlation between plasma lipid levels and multiple anthropometric and metabolic parameters was assessed by Pearson’s correlation coefficients. To examine the discriminative power and accuracy of any anthropometric parameter for dyslipidemia, the area under receiver operating characteristics (ROC) curves were calculated. All these statistical analysis were performed using SPSS 20.0 (IBM Corp. 2011).

## Results

### Plasma lipids levels are correlated with various anthropometric and metabolic parameters

We first determined the correlation between different anthropometric/metabolic variables and plasma lipid levels (Table 1). Plasma HDL levels showed very weak/weak negative but statistically significant correlation with multiple variables including body weight, BMI, body fat percentage, total body fat mass, waist circumference, wrist circumference, waist-to-height ratio, BRI, RMR, and systolic and diastolic blood pressure. On the other hand, plasma LDL levels displayed very weak/weak positive and statistically significant correlation with body weight, BMI, body fat percentage, total body fat mass, waist circumference, wrist circumference, waist-to-height ratio, and BRI.

**Table 1:** Pearson’s correlation coefficients between plasma lipid levels and various anthropometric/metabolic parameters

Anthropometric/metabolic parameter	HDL	LDL	TG
Body weight	−0.220**	0.160*	0.316**
BMI	−0.183**	0.248**	0.240**
Body fat	−0.163*	0.235**	0.339**
Total body fat mass	−0.192**	0.195**	0.327**
Waist circumference	−0.191**	0.207**	0.350**
Wrist circumference	−0.187**	0.140*	0.364**
Waist-to-hip ratio	−0.047	0.082	0.252**
Waist-to-height ratio	−0.169**	0.246**	0.302**
ABSI	−0.44	0.021	0.205**
BRI	−0.170**	0.241**	0.271**
Age	−0.048	0.105	0.345**
RMR	−0.215**	0.030	0.155*
Fasting glucose	−0.084	0.091	0.303**
Systolic BP	−0.152*	0.014	0.200**
Diastolic BP	0.175**	0.062	0.172**

*Correlation is significant at the 0.05 level; **correlation is significant at the 0.01 level. *Abbreviations*: *BMI* Body mass index, *ABSI* a body shape index, *BRI* body roundness index, *RMR* resting metabolic rate, *BP* blood pressure

Nevertheless, plasma TG levels showed weak positive and statistically significant correlation with all the anthropometric/metabolic variables studied for the present work (Table 1). Here, the values for the correlation coefficient were slightly higher than that in the aforementioned LDL and HDL analyses.

### Multiple anthropometric and metabolic parameters significantly vary with severity and patterns of dyslipidemia

**Fig. 1:**
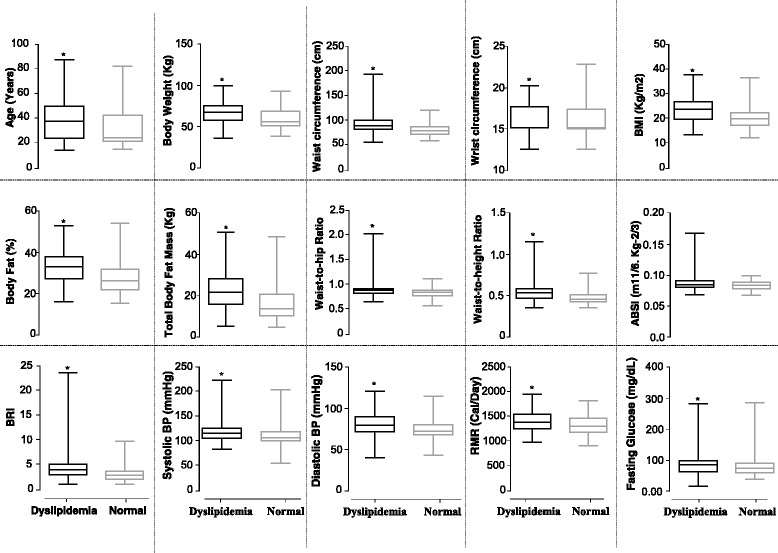
Comparison of various anthropometric and metabolic parameters in dyslipidemic versus non-dyslipidemic participants. Box-plot representation of the values for different anthropometric/metabolic parameters in dyslipidemic and non-dyslipidemic participants. Dyslipidemia was defined as irregularity in the plasma levels of HDL, LDL, or Triglycerides. *Boxes* represent median and interquartile range; *whiskers* represent minimum and maximum. Significance was determined by unpaired *t* test. *Significant difference (*p* < 0.01)

We compared various anthropometric and metabolic parameters between dyslipidemic and non-dyslipidemic participants. As shown in Fig. 1, the dyslipidemic participants display significantly higher values for all anthropometric and metabolic variables analyzed in the present study except ABSI.

**Fig. 2:**
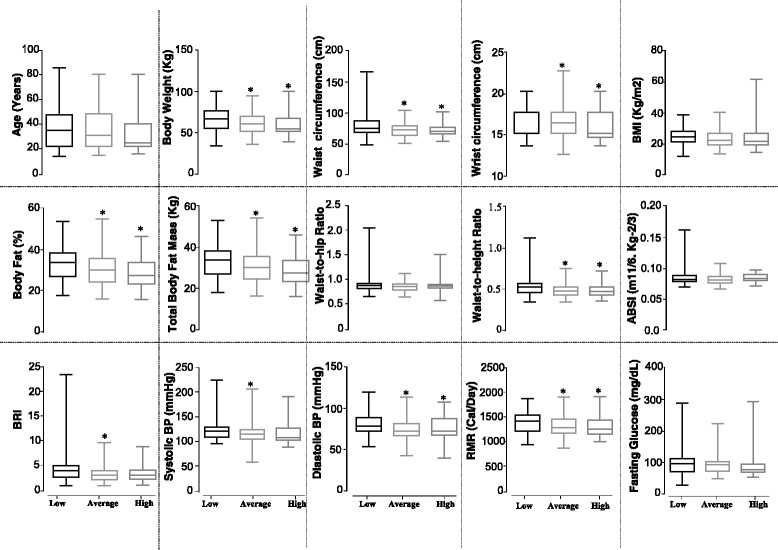
Effects of occurrence and severity of plasma HDL aberrations on various anthropometric/metabolic parameters. Box-plot representation of the values for different anthropometric/metabolic parameters in different subgroups of the study population classified according to plasma HDL levels—i.e., low-, average, and high-HDL group (Additional file 2: Table S2). *Boxes* represent median and interquartile range; *whiskers* represent minimum and maximum. Significance was determined by unpaired *t* test. Symbol of statistical significance (*) refers to the comparison of low-HDL vs. average-HDL and low-HDL vs. high-HDL (*p* < 0.01)

Next, we sought to determine the effect of type and severity of dyslipidemia on various anthropometric and metabolic parameters. Figure 2 displays the variations in multiple parameters in different subgroups of the study population classified according to plasma HDL levels, i.e., average, low-, and high-HDL group. The low-HDL group displays significantly higher values for various parameters—including body weight, waist circumference, wrist circumference, body fat percentage, total body fat mass, waist-to-height ratio, diastolic blood pressure, and RMR—in comparison to the average or high-HDL groups. Moreover, the low-HDL group displays significantly higher values for BRI and systolic blood pressure only in comparison to the average HDL group, but not the high-HDL group. No significant difference was observed between the average and high-HDL groups for any of the tested parameters.

**Fig. 3:**
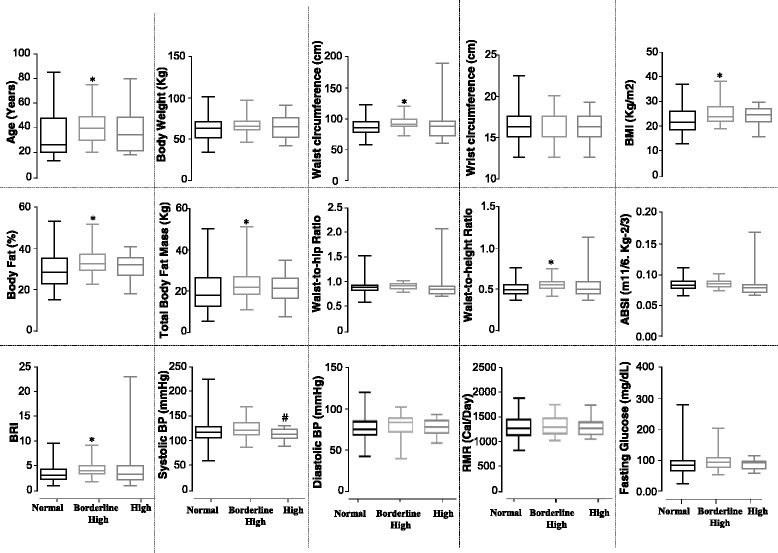
Effects of occurrence and severity of plasma LDL aberrations on various anthropometric/metabolic parameters. Box-plot representation of the values for different anthropometric/metabolic parameters in different subgroups of the study population classified according to plasma LDL levels—i.e., normal-, borderline high-, and high-LDL group (Additional file 2: Table S2). *Boxes* represent median and interquartile range; *whiskers* represent minimum and maximum. Significance was determined by unpaired *t* test. Symbol of statistical significance (*) and (#) refer to the comparison of normal-LDL vs. borderline high-LDL and borderline high- vs. high-LDL group, respectively (*p* < 0.01)

Next, we compared various anthropometric and metabolic parameters in the different subgroups of the study population categorized according to plasma LDL levels. It was observed that the values for age, waist circumference, BMI, body fat percentage, total body fat mass, waist-to-height ratio, and BRI were significantly higher in the borderline high-LDL group than in the normal-LDL group (Fig. 3). Systolic blood pressure displayed significant difference only between the borderline high- and high-LDL groups.

**Fig. 4:**
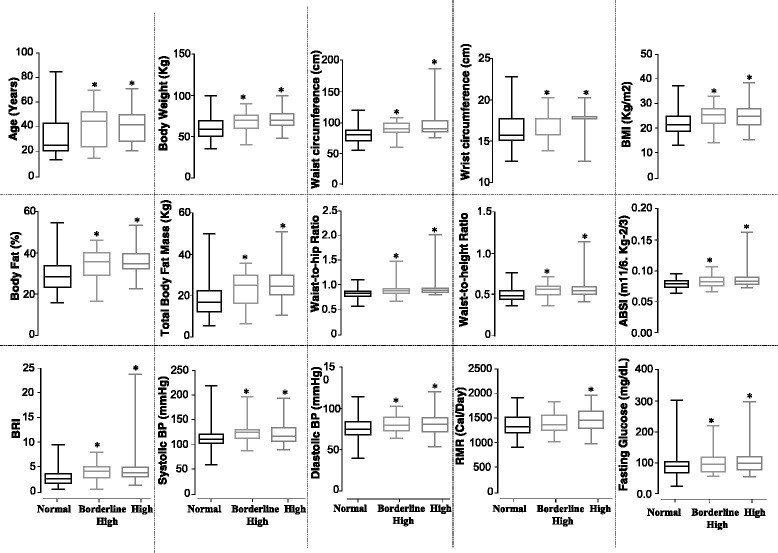
Effects of occurrence and severity of hypertriglyceridemia on various anthropometric/metabolic parameters. Box-plot representation of the values for different anthropometric/metabolic parameters in different subgroups of the study population classified according to plasma triglyceride (TG) levels—i.e., normal-, borderline high-, and high-TG group (Additional file 2: Table S2). *Boxes* represent median and interquartile range; *whiskers* represent minimum and maximum. Significance was determined by unpaired *t* test. Symbol of statistical significance (*) refers to the comparison of normal-TG vs. borderline high TG and normal-TG vs. high-TG (*p* < 0.01)

Next, we assessed the differences in various anthropometric and metabolic parameters between the different TG subgroups within our study population, i.e., normal-, borderline high-, and high-TG groups. The borderline high- and high-TG group displayed significantly high values for all the tested variables in comparison to normal-TG group, except RMR, that only displayed a significant increase in the high-TG group than in the normal-TG group (Fig. 4). No significant difference was observed between the borderline high- and high-TG group for any of the parameters analyzed for the presented study.

### Predictive capabilities of multiple anthropometric/metabolic parameters fluctuate for different types of dyslipidemias

**Fig. 5:**
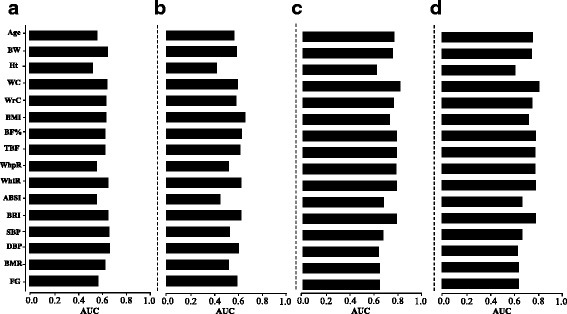
The capability of various anthropometric/metabolic variables to predict the absence or presence of different forms of dyslipidemia. *Bar graphs* represent the values for area under the curves from receiver operating characteristic (ROC) curve analysis to determine the predictive capability of different anthropometric/metabolic parameters for identification of **a** overall dyslipidemia (irregularity in the plasma levels of HDL, LDL, or Triglycerides), **b** low-HDL levels, **c** high-LDL, and **d** high-TG. Abbreviations: *BW* Body weight, *Ht* Height, *WC* waist circumference, *WrC* Wrist circumference, *BMI* Body mass index, *BF%* Body fat percentage, *TBF* Total body fat mass, *WhpR* Waist-to-hip ratio, *WhtR* Waist-to-height ratio, *ABSI* A body shape index, *BRI* Body roundness index, *SBP* Systolic blood pressure, *DBP* Diastolic blood pressure, *RMR* Resting metabolic rate, and *FG* Fasting glucose

We performed receiver operating characteristic (ROC) curve analysis to check the predictive capability of different anthropometric/metabolic parameters for distinguishing between dyslipidemic and non-dyslipidemic individuals. Waist circumference, waist-to-height ratio, and BRI gave the highest and similar values (~0.71) for area under the curve (AUC) to predict dyslipidemia (Fig. 5, Additional file 1: Figure S3 and Additional file 2: Table S3). Moreover, the AUC values of body weight, BMI, body fat percentage, total body fat mass, wrist circumference, waist-to-hip ratio, and age were also moderately high (0.61–0.68).

The predictive capabilities of the corresponding parameters in distinguishing the participants with high HDL from that with low/average HDL were also analyzed. It was observed that various parameters—including body weight, BMI, body fat percentage, total body fat mass, waist circumference, wrist circumference, waist-to-height ratio, BRI, RMR, and systolic and diastolic BP—showed moderately high and similar (0.61–0.64) AUC values in predicting low/average HDL levels (Fig. 5, Additional file 1: Figure S3 and Additional file 2: Table S3). Nevertheless, systolic and diastolic BP gave the highest AUC values in these analyses.

Comparatively fewer parameters showed moderate discriminatory power in distinguishing the participants with normal-LDL levels from that with borderline high-/high-LDL levels. These parameters included BMI, body fat percentage, total body fat mass, waist-to-height ratio, and BRI (AUC ~0.61–0.64). Here, BMI showed the highest AUC value (0.645) (Fig. 5, Additional file 1: Figure S3 and Additional file 2: Table S3).

All the tested parameters showed the highest predictive capabilities for detecting borderline/high-TG levels. Body-weight, BMI, body fat percentage, total body fat mass, waist circumference, wrist circumference, waist-to-hip ratio, waist-to-height ratio, BRI, and age gave high-AUC values (0.70–0.78) in predicting borderline/high-TG levels. Moreover, RMR, fasting glucose levels, and systolic and diastolic BP also gave moderately high-AUC values (0.60–0.65) (Fig. 5, Additional file 1: Figure S3 and Additional file 2: Table S3). ABSI, which gave very low AUC values in predicting any of the aforementioned dyslipidemias, also gave a moderately high-AUC value (0.64) in predicting borderline/high-TG levels. These ROC curve analyses revealed that the discriminatory power of the most of tested anthropometric and metabolic parameters were the highest for detecting hypertriglyceridemia.

## Discussion

The major goal of the presented work was to examine the relationship between different anthropometric indices and dyslipidemia. We first studied the correlation between plasma lipid levels and different anthropometric and metabolic indices. In accordance with the previous studies [2–6], various body indices including the classical indicators of obesity—BMI and waist circumference—showed a statistically significant but weak positive correlation with serum cholesterol, LDL and TG levels [2–6], whereas a weak negative correlation with serum HDL levels.

A previous study investigated the effects of severity of hypercholesterolemia (high plasma LDL levels) on BMI [34]. They reported that higher BMI category was associated with higher LDL levels in school children [34]. Here, we performed detailed analyses for demonstrating the impact of incidence and type and severity of dyslipidemia on multiple anthropometric and metabolic indices. For these analyses, the study population was classified into different subgroups that included subgroups of the participants with normal lipid profiles or with different degrees of dyslipidemia. When dyslipidemic participants (displaying irregularity in the plasma levels of at least one of the major lipoprotein fractions) were compared with non-dyslipidemic participants, significant differences were observed for almost all of the studied anthropometric parameters. Next, we determined the effect of different types and degrees of dyslipidemia on corresponding parameters. It was observed that the low-HDL group displays significantly higher values for various anthropometric parameters in comparison to average or high-HDL groups. In the subgroups categorized according to plasma LDL levels, the major differences were observed between the borderline high- and normal-LDL groups. Nevertheless, borderline high- and high-TG group displayed significantly high values for almost all the tested variables in comparison to the normal-TG group. These data indicate that the major differences were observed between subgroups with or without the different forms of dyslipidemia. Conversely, the anthropometric variables may not give sharp distinction among subgroups with different degrees of dyslipidemia.

It is noteworthy that the correlation analyses between plasma TG levels and various anthropometric parameters gave the highest values for Pearson’s correlation coefficients. Moreover, the subgroups categorized according to plasma TG levels gave significant differences for all the tested anthropometric variables. Further, analyses revealed that several anthropometric/metabolic indices display increased predictive capabilities for detecting hypertriglyceridemia in comparison to detecting any other form of plasma lipid disorders. This observation is also in accordance with a previous study that gave higher predictive capabilities of BMI, waist-to-hip ratio, and waist circumference for hypertriglyceridemia [35].

In the present study, we also evaluated the significance of recently described body indices, ABSI and BRI, in assessment of incidence and type and severity of dyslipidemia. We observed that the capacity of BRI to predict dyslipidemia was comparable but not superior to the classical indicators of obesity—including BMI, body fat percentage, and waist circumference—whereas ABSI could not detect the presence or absence of dyslipidemia. Previous reports provide contradictory data on significance of ABSI in determining the health status of adolescents. ABSI was initially reported to predict premature mortality better than BMI or WC [21]. However, recent study showed that ABSI cannot distinguish between individuals with and without CVD or CVD risk factors, including dyslipidemia [27]. The same study also reported that the capacity of BRI as a novel body index to identify CVD was not superior in comparison to the established anthropometric indices such as BMI and WC. Our data also confirms these previous observations.

## Conclusions

The present work revisits the association between plasma lipid levels and different anthropometric indices. The significance of these body indices in clinical assessment of type and severity of dyslipidemia was also determined. We observed significant changes in anthropometric measurements with incidence and severity of dyslipidemia. The predictive capabilities of various anthropometric/metabolic indices for different forms of dyslipidemia were also compared. It was observed that all the body indices display the highest predictive capabilities for detecting hypertriglyceridemia in comparison to any other form of plasma lipid disorders. We also studied the significance of recently described body indices, ABSI and BRI, in assessment of dyslipidemia. The capacity of BRI to predict dyslipidemia was comparable but not superior to the classical indicators of obesity, whereas ABSI could not detect dyslipidemia. The presented study contributes to the existing knowledge on association between anthropometric/metabolic variables and plasma lipid levels. This data may have implications in the diagnosis and characterization of dyslipidemia as well as for the assessment of related disease risks.

## Additional files

Additional file 1: Figure S1. Enrolment flowchart of the study population. Two hundred seventy-five participants visited the free health camps. Thirty-seven participants were excluded because of HCV diagnosis (*n*=5), Postprandial state (*n*=30) and diabetes (*n*=2). The final group sample consisted of 238 participants. Figure S2: Venn-Diagram displays overlaps between prevalence of TG, high-LDL, and low-HDL levels in the dyslipidemic population. Figure S3: Receiver operating characteristic (ROC) curve analysis to determine the predictive capability of different anthropometric/metabolic parameters for identification of (a) overall dyslipidemia (irregularity in the plasma levels of HDL, LDL, or triglycerides, (b) low-HDL levels, (c) high-LDL, and (d) high-TG. (PPT 370 kb)
Additional file 2: Table S1. Clinical and metabolic characteristics of the study population. Table S2: Criteria for the determination of plasma lipoprotein/ lipid abnormality status. Table S3: Areas under ROC curves (AUC) and 95% confidence intervals (CI) for various anthropometric/metabolic parameters in predicting the incidence of overall dyslipidemia or different types of dyslipidemias. Results are represented as AUC (95% CI). (DOC 795 kb)

## Abbreviations

AUC: Area under the curve
HDL: High-density lipoprotein cholesterol
LDL: Low-density lipoprotein cholesterol
ROC: Receiver Operating Characteristics
TC: Total cholesterol
