# Correction to: Anthropometric and metabolic indices in assessment of type and severity of dyslipidemia

**DOI:** 10.1186/s40101-017-0155-5

**Published:** 2017-11-22

**Authors:** Muhammad Zaid, Fatima Ameer, Rimsha Munir, Rida Rashid, Nimrah Farooq, Shahida Hasnain, Nousheen Zaidi

**Affiliations:** Microbiology and Molecular Genetics, University of the Punjab, Lahore, 54590 Pakistan

## Correction

After the publication of this work [1] an error was noticed in one of the formulas. Due to an oversight, a typographical error was identified in the formula for body roundness index (BRI) on page 3 of the article. The printed formula is:

$$ Body Roundness Index\;(BRI)=364.2-365.5\times \sqrt{1-\left(\frac{\left( WC/{\left(2\pi \right)}^2\right)}{{\left(0.5\;x\; Height\right)}^2}\right)} $$

However, the correct formula for BRI is as follows [2–4].

$$ Body Roundness Index\;(BRI)=364.2-365.5\times \sqrt{1-\left(\frac{{\left( WC/\left(2\pi \right)\right)}^2}{{\left(0.5\;x\; Height\right)}^2}\right)} $$
