# Supplementary material for: Anthropometric and metabolic indices in assessment of type and severity of dyslipidemia
Source: J Physiol Anthropol. 2017 Feb 28;36:19. doi: 10.1186/s40101-017-0134-x (PMC5330152; doi:10.1186/s40101-017-0134-x)
Supplement: Additional file 1: Figure S1. — Enrolment flowchart of the study population. Two hundred seventy-five participants visited the free health camps. Thirty-seven participants were excluded because of HCV diagnosis (n=5), Postprandial state (n=30) and diabetes (n=2). The final group sample consisted of 238 participants. Figure S2: Venn-Diagram displays overlaps between prevalence of TG, high-LDL, and low-HDL levels in the dyslipidemic population. Figure S3: Receiver operating characteristic (ROC) curve analysis to determine the predictive capability of different anthropometric/metabolic parameters for identification of (a) overall dyslipidemia (irregularity in the plasma levels of HDL, LDL, or triglycerides, (b) low-HDL levels, (c) high-LDL, and (d) high-TG. (PPT 370 kb) [file 40101_2017_134_MOESM1_ESM.ppt]

## Slide 1
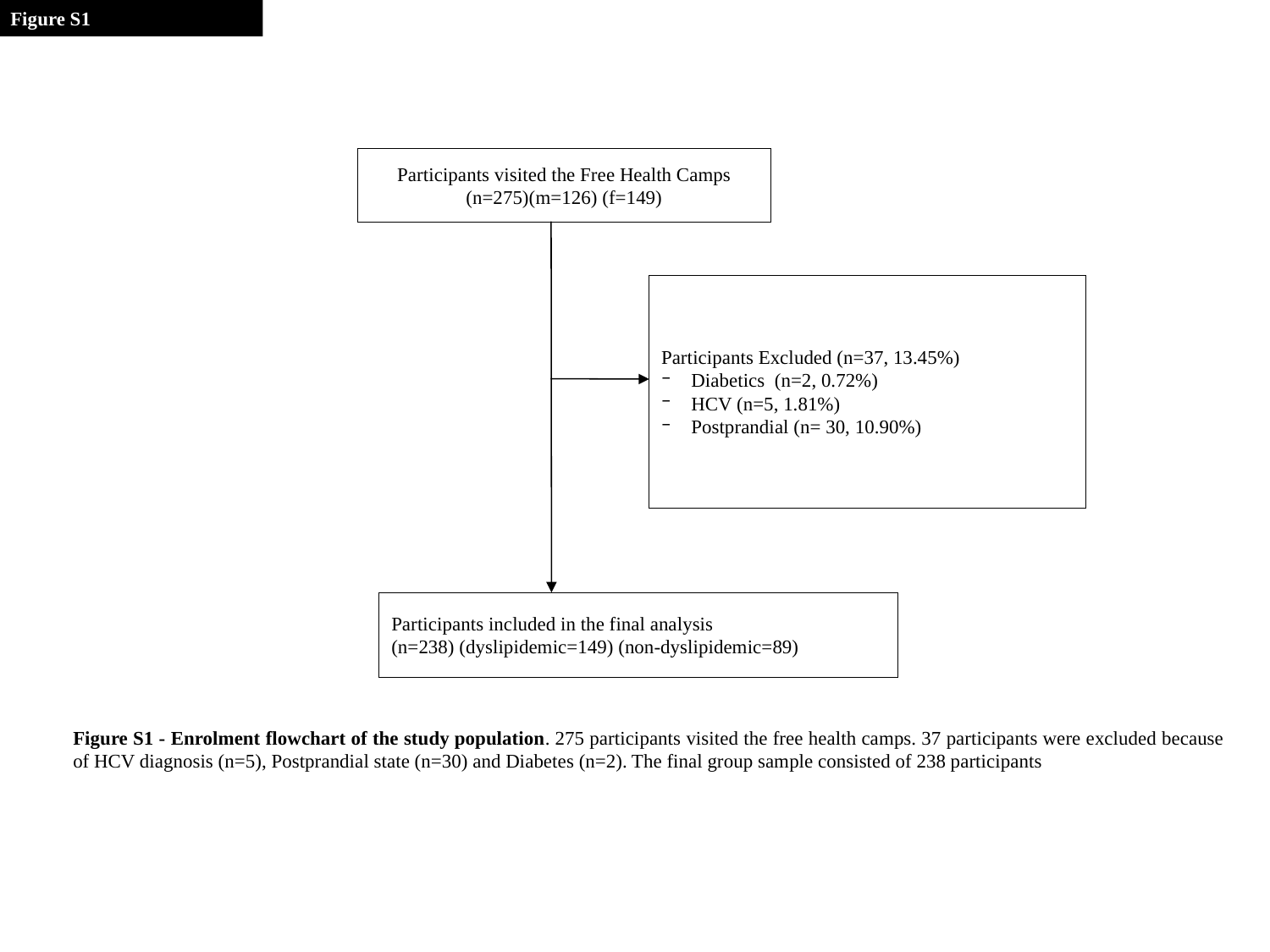

Figure S1
Participants visited the Free Health Camps (n=275)(m=126) (f=149)
Participants Excluded (n=37, 13.45%)
Diabetics (n=2, 0.72%)
HCV (n=5, 1.81%)
Postprandial (n= 30, 10.90%)
Participants included in the final analysis
(n=238) (dyslipidemic=149) (non-dyslipidemic=89)
Figure S1 - Enrolment flowchart of the study population. 275 participants visited the free health camps. 37 participants were excluded because of HCV diagnosis (n=5), Postprandial state (n=30) and Diabetes (n=2). The final group sample consisted of 238 participants

## Slide 2
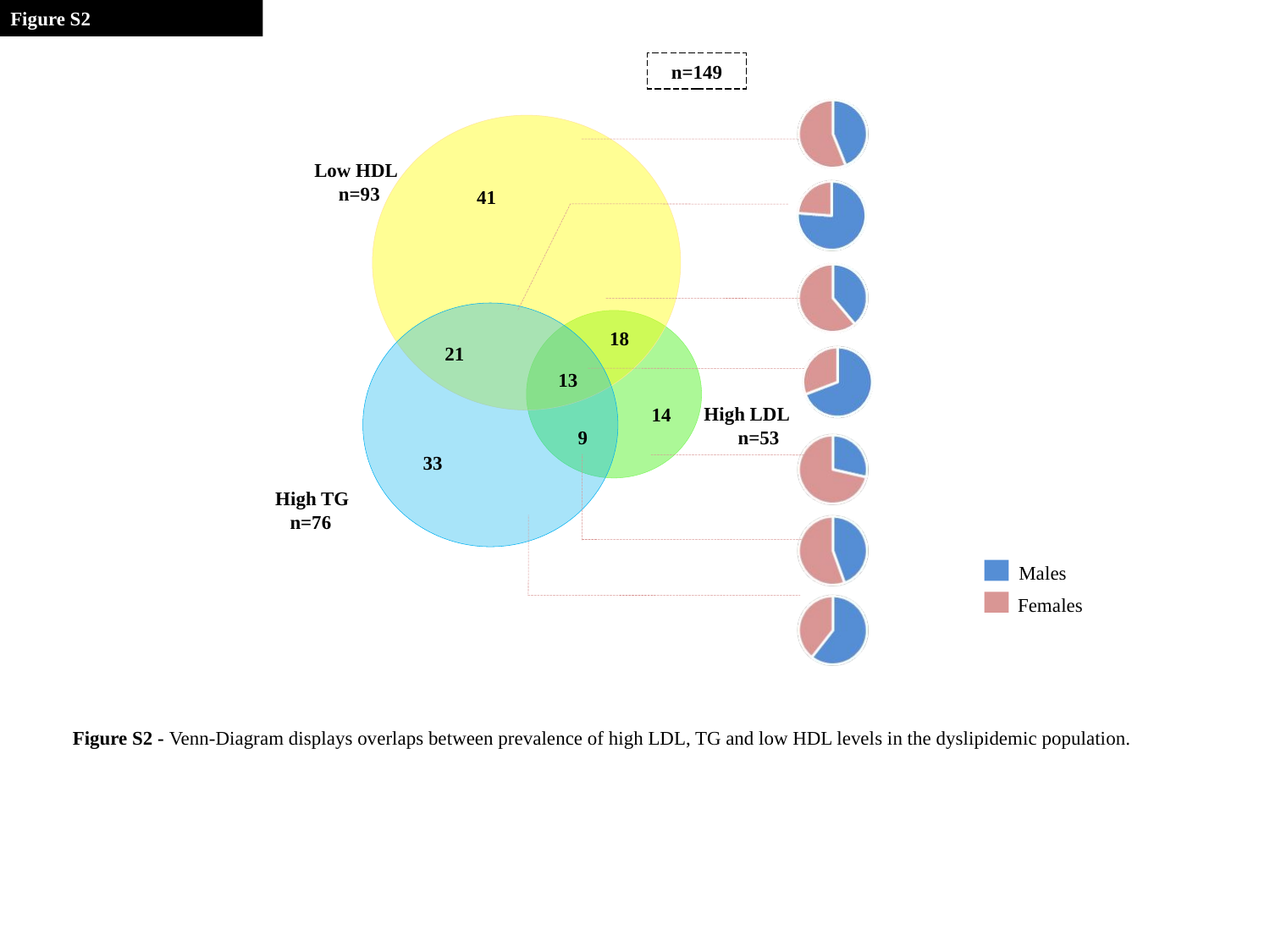

Figure S2
n=149
Low HDL
 n=93
41
18
21
13
High LDL
 n=53
14
9
33
High TG
 n=76
Males
Females
Figure S2 - Venn-Diagram displays overlaps between prevalence of high LDL, TG and low HDL levels in the dyslipidemic population.

## Slide 3
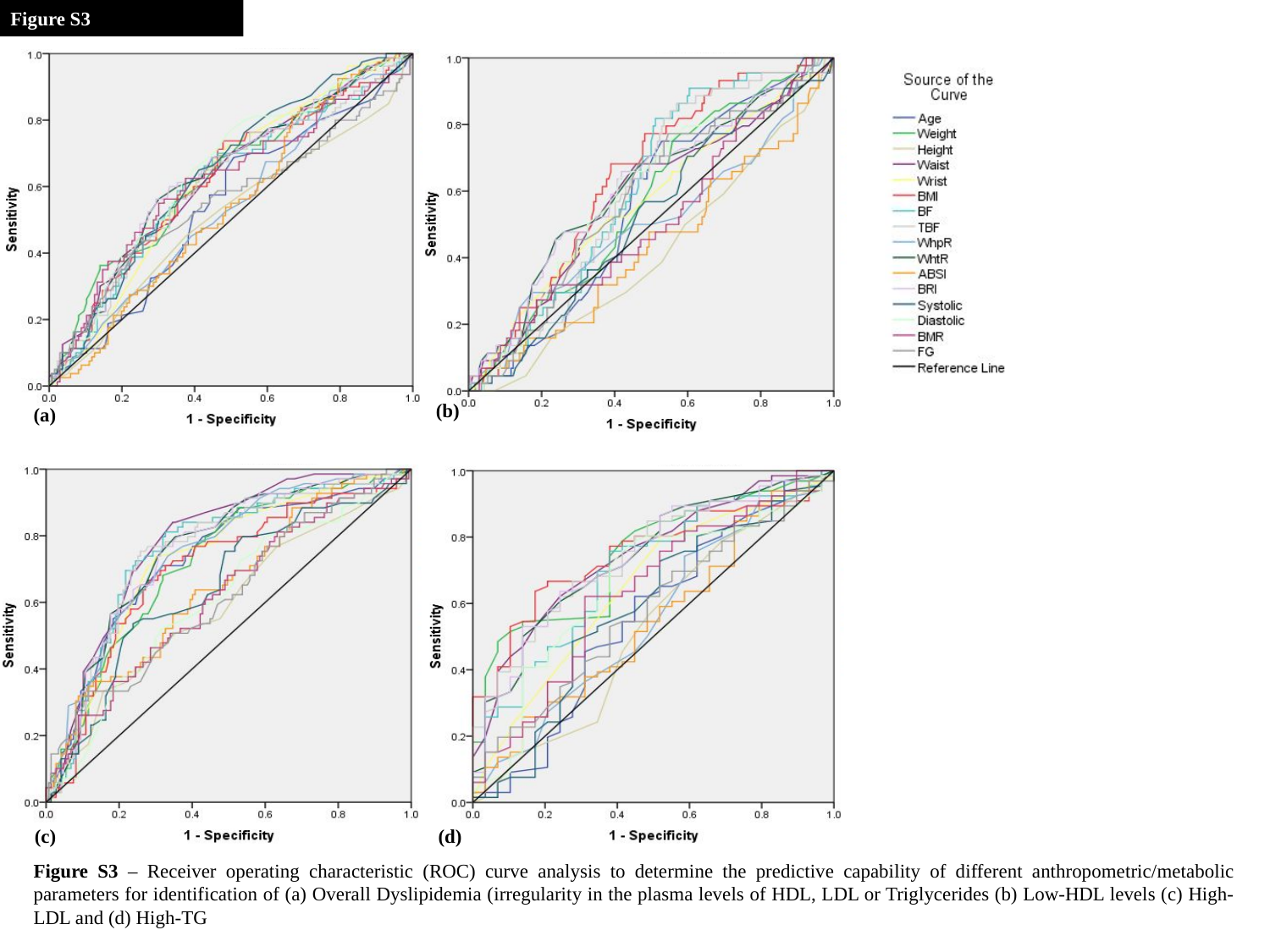

Figure S3
(b)
(a)
(b)
(a)
(d)
(e)
(c)
(d)
Figure S3 – Receiver operating characteristic (ROC) curve analysis to determine the predictive capability of different anthropometric/metabolic parameters for identification of (a) Overall Dyslipidemia (irregularity in the plasma levels of HDL, LDL or Triglycerides (b) Low-HDL levels (c) High-LDL and (d) High-TG
