# Supplementary material for: Anthropometric and metabolic indices in assessment of type and severity of dyslipidemia
Source: J Physiol Anthropol. 2017 Feb 28;36:19. doi: 10.1186/s40101-017-0134-x (PMC5330152; doi:10.1186/s40101-017-0134-x)
Supplement: Additional file 2: Table S1. — Clinical and metabolic characteristics of the study population. Table S2: Criteria for the determination of plasma lipoprotein/ lipid abnormality status. Table S3: Areas under ROC curves (AUC) and 95% confidence intervals (CI) for various anthropometric/metabolic parameters in predicting the incidence of overall dyslipidemia or different types of dyslipidemias. Results are represented as AUC (95% CI). (DOC 795 kb) [file 40101_2017_134_MOESM2_ESM.doc]

**Table S1-** Clinical and metabolic characteristics of the study population

| **Parameter** | **Non-Dyslipidemic Controls**  **n=89** | **Dyslipidemics**  **n=149** | **p-values** |
| --- | --- | --- | --- |
| Weight, Kg | 58.22±11.98 | 67.03±13.08 | ****< 0.0001 |
| BMI, Kg/m^2^ | 21.78±4.57 | 24.67±4.67 | ****<0.0001 |
| Body Fat, % | 27.98±7.60 | 33.06±7.48 | ****<0.0001 |
| Total Body Fat Mass, Kg | 17.08±8.37 | 22.83±8.86 | ****<0.0001 |
| Waist Circumference, cm | 79.87±12.31 | 89.30±15.32 | ****<0.0001 |
| Wrist Circumference, cm | 15.98±1.85 | 16.99±1.67 | ****<0.0001 |
| Waist-to-hip Ratio | 0.84±0.08 | 0.88±0.13 | **0.0017 |
| Waist-to-height Ratio | 0.49±0.08 | 0.54±0.09 | ****<0.0001 |
| ABSI (m^11/6^. Kg^-2/3^) | 0.08±0.01 | 0.08±0.01 | 0.0866 |
| BRI | 3.26±1.53 | 4.39±2.29 | ****<0.0001 |
| Age, years | 31.61±15.10 | 38.46±16.26 | ** 0.0012 |
| RMR (Cal/Day) | 1338.50±182.34 | 1416.16±207.16 | **0.0021 |
| Fasting Glucose (mg/dL) | 84.52±30.57 | 95.75±42.41 | *0.0252 |
| Systolic BP (mmHg) | 117.08±20.31 | 122.81±19.90 | *0.0331 |
| Diastolic BP (mmHg) | 73.98±12.04 | 79.35±12.31 | ***0.0007 |
| TC (mg/dL) | 136.49±35.21 | 177.52±55.45 | ****<0.0001 |
| LDL (mg/dL) | 62.13±32.63 | 112.83±52.15 | ****<0.0001 |
| HDL (mg/dL) | 56.72±14.38 | 37.32±13.55 | ****<0.0001 |
| TG (mg/dL) | 90.48±30.19 | 170.54±98.97 | ****<0.0001 |
| Male: Female Ratio | 30:59 | 78:71 |  |
| Urban: Rural Ratio | 68:21 | 103:46 |  |
| Smokers (%) | 7.86 | 15.43 |  |

Data is represented as mean ± S.D except for male: female ratio, urban: rural ratio and smokers’ percentage. *correlation is significant at the 0.05 level; **correlation is significant at the 0.01 level, ***correlation is significant at the 0.001 level; ****correlation is significant at the 0.0001 level. Abbreviations: BMI; Body mass index, ABSI; a body shape index, BRI; body roundness index, RMR; resting metabolic rate, BP; blood pressure, TC; total cholesterol, LDL; low-density lipoprotein, HDL; high-density lipoprotein, TGs; triglycerides.

**Table S2:** Criteria for the determination of plasma lipoprotein/ lipid abnormality status.

| **HDL (mg/dL)** | |
| --- | --- |
| Low | <40 |
| Average | 40-60 |
| High | >60 |
| **LDL (mg/dL)** | |
| Normal | <100 - 129 |
| Borderline high | 130 - 159 |
| High - Very High | 160 - ≥190 |
| **Triglycerides (mg/dL)** | |
| Normal | <150 |
| Borderline high | 150-199 |
| High-Very High | 200- >500 |

Abbreviations: HDL; high-density lipoprotein, LDL; low-density lipoprotein, TGs; triglycerides.

**Table S3:** Areas under ROC curves (AUC) and 95% confidence intervals (CI) for various anthropometric/metabolic parameters in predicting the incidence of overall dyslipidemia or different types of dyslipidemias. Results are represented as *AUC (95% CI)*.

| **Predicting Parameter** | **Type of Dyslipidemia** | | | |
| --- | --- | --- | --- | --- |
|  | **Dyslipidemia** | **Low HDL levels** | **Borderline High/High LDL Levels** | **Borderline High/High TG Levels** |
| Body Weight | 0.688 (0.616-0.759) | 0.630 (0.553-0.707) | 0.574 (0.486-0.662) | 0.722 (0.651-0.793) |
| BMI | 0.694 (0.622-0.766) | 0.619 (0.541-0.696) | 0.645 (0.563-0.727) | 0.700 (0.624-0.777) |
| Body Fat % | 0.698 (0.626-0.770) | 0.610 (0.531-0.689) | 0.613 (0.531-0.695) | 0.756 (0.686-0.825) |
| Total Body Fat Mass | 0.699 (0.628-0.770) | 0.612 (0.533-0.690) | 0.604 (0.521-0.687) | 0.753 (0.683-0.822) |
| Waist Circumference | 0.712 (0.642-0.782) | 0.628 (0.552-0.705) | 0.581 (0.485-0.676) | 0.784 (0.721-0.846) |
| Wrist Circumference | 0.679 (0.606-0.753) | 0.621 (0.546-0.696) | 0.570 (0.474-0.666) | 0.729 (0.658-0.800) |
| Waist-to-hip Ratio | 0.625 (0.549-0.700) | 0.541 (0.462-0.620) | 0.508 (0.406-0.610) | 0.752 (0.685-0.820) |
| Waist-to-height Ratio | 0.710 (0.640-0.780) | 0.637 (0.560-0.713) | 0.612 (0.517-0.706) | 0.756 (0.689-0.823) |
| ABSI | 0.559 (0.480-0.637) | 0.540 (0.463-0.618) | 0.437 (0.340-0.535) | 0.649 (0.571-0.727) |
| BRI | 0.710 (0.640-0.780) | 0.637 (0.560-0.714) | 0.610 (0.516-0.705) | 0.756 (0.688-0.823) |
| Age | 0.621 (0.544-0.699) | 0.544 (0.464-0.623) | 0.556 (0.471-0.641) | 0.733 (0.661-0.806) |
| RMR | 0.606 (0.530-0.682) | 0.613 (0.533-0.694) | 0.511 (0.415-0.607) | 0.617 (0.537-0.698) |
| Fasting Glucose | 0.584 (0.507-0.660) | 0.556 (0.472-0.640) | 0.579 (0.488-0.670) | 0.618 (0.537-0.699) |
| Systolic BP | 0.622 (0.546-0.699) | 0.644 (0.569-0.718) | 0.518 (0.427-0.609) | 0.646 (0.567-0.726) |
| Diastolic BP | 0.652 (0.577-0.726) | 0.646 (0.571-0.722) | 0.590 (0.498-0.683) | 0.609 (0.528-0.690) |

Abbreviations: BMI; Body mass index, ABSI; a body shape index, BRI; body roundness index, RMR; resting metabolic rate, BP; blood pressure.
